# Understanding the hidden burden: prevalence and factors linked to left ventricular hypertrophy in hypertensive patients receiving care in Harari region, Ethiopia

**DOI:** 10.3389/fcvm.2025.1533707

**Published:** 2025-04-17

**Authors:** Ashenafi Tadesse, Zerihun Abera Ayele, Megnot Hailu Mekonnen, Sinetibeb Mesfin, Dawit Abebe

**Affiliations:** ^1^ School Medicine, College of Medicine and Health Sciences, Haramaya University, Harar, Ethiopia; ^2^ School of Medicine, College of Health and Medical Sciences, Jigjiga University, Jigjiga, Ethiopia; ^3^ School of Nursing and Midwifery, College of Health and Medical Sciences, Jigjiga University, Jigjiga, Ethiopia; ^4^ School of Nursing and Midwifery, College of Medicine and Health Sciences, Haramaya University, Harar, Ethiopia

**Keywords:** hypertension, left ventricular hypertrophy, echocardiography, Ethiopia, eastern Ethiopia

## Abstract

**Background:**

Left ventricular hypertrophy (LVH) is often a complication of hypertension and an independent risk factor for cardiovascular events. In Ethiopia, there is a scarcity of data on the prevalence and associated factors of left ventricular hypertrophy among hypertensive adults. This study aimed to assess the prevalence and associated factors of left ventricular hypertrophy among adult patients with hypertension attending treatment at two public hospitals in Harar, Eastern Ethiopia from 20 December 2021 to 20 December 2023.

**Method:**

A hospital-based cross-sectional study was conducted on 264 hypertensive patients from 20 December 2021 to 20 December 2023. A pretested structured questionnaire and checklist were used to collect data from participants and their clinical records. The data were collected by trained residents and interns. Data were analyzed using SPSS version 29. Left ventricular mass was measured by transthoracic echocardiography. Associations between categorical variables were assessed using a chi-square test and odds ratio with 95% confidence interval. A logistic regression model was used to identify risk factors of LVH. *p*-values of <0.05 were considered as statistically significant.

**Results:**

The study included 264 adults with hypertension, with a mean age of 58.4 years, and the majority (54.5%) were male. The prevalence of echocardiographically confirmed left ventricular hypertrophy (LVH) was 30.7% (95% CI: 25.1%–36.3%), with mild LVH being the most common type (51%). Significant predictors of LVH included age over 60 years [adjusted odds ratio (AOR) = 5.981, CI = 1.832–19.522, *p* = 0.003], khat chewing (AOR = 2.676, CI = 1.786–9.109, *p* = 0.001), diabetes (AOR = 10.430, CI = 2.904–37.454, *p* < 0.001), poor medication adherence (AOR = 4.132, CI = 1.208–14.141, *p* = 0.024), uncontrolled systolic blood pressure (AOR = 8.340, CI = 2.280–30.512, *p* = 0.001), lack of home blood pressure monitoring (AOR = 5.591, CI = 1.041–30.012, *p* = 0.045), and longer hypertension duration (AOR = 8.766, CI = 2.101–36.584, *p* = 0.003). These findings emphasize the need to address modifiable risk factors in managing hypertension to reduce the burden of LVH.

**Conclusions:**

The echocardiographic prevalence of LVH was 30.7% in the study population. These results highlight the importance of addressing modifiable risks to reduce LVH burden.

## Background

Hypertension is defined as an office systolic blood pressure (SBP) value of at least 140 mmHg and/or diastolic BP (DBP) value of at least 90 mmHg. The relationship between blood pressure and cardiovascular and renal events is continuous, making the distinction between normotension and hypertension, based on cutoff blood pressure values, somewhat arbitrary (1). More than a quarter of the world's population had hypertension in 2000, and by 2025, it may increase to 1.56 billion.

The World Health Organization (WHO) estimated that approximately 62% of cardiovascular diseases (CVDs) and 49% of ischemic heart diseases are attributable to high BP in the world (2). It has been estimated that one out of four adults worldwide (1.39 billion people) have hypertension, and this rate is expected to increase as a result of various epidemiological and demographic factors, such as urbanization, especially in low- and middle-income countries. Approximately 74.7 million individuals have hypertension in sub-Saharan Africa (SSA), and this number is expected to reach 125.5 million individuals by 2025 (3). Hypertension has become an important chronic non-communicable disease with increasing trends in developing countries, and uncontrolled BP will result in life-threatening complications (4).

Other than regional studies in different parts of the country, there are no well-designed studies on the national prevalence of hypertension in Ethiopia. According to a systematic meta-analysis, the estimated prevalence of hypertension among the Ethiopian population is 19.6% with hypertension being higher in urban (23.7%) than in rural areas and among males (20.6%) than females (19.2%) (5, 6). A systematic review of the epidemiology of hypertension in Ethiopia (2020) reported varying prevalence rates across different study types. Population-based studies found hypertension prevalence to range from 9.3% to 30.3%, while institution-based studies reported rates between 7% and 37%. Hospital-based studies revealed a prevalence of 13.2%–18.8% (7).

Left ventricular hypertrophy (LVH) is an anatomic enlargement and thickening of the left ventricle. LVH in hypertension is a structural change and a physiological adaptation of the left ventricular myocardium as a result of increased workload on the left ventricular chamber (8, 9). Left ventricular hypertrophy is classified as eccentric or concentric hypertrophy. Concentric hypertrophy results from a steady state of pressure overload as occurs in a state of longstanding hypertension or aortic stenosis. It is characterized by an increased ratio of wall thickness to chamber dimension and is associated with a worse prognosis than eccentric hypertrophy. Eccentric hypertrophy results from a longstanding state of volume overload as occurs aortic or mitral regurgitation. It is characterized by an increased ratio of chamber dimension to wall thickness (10–12). There are various diagnostic modalities for the detection of LVH. These include electrocardiography (ECG), two- or three-dimensional echocardiography (2/3D ECHO), and magnetic resonance imaging (MRI). Echocardiography has been the gold standard for the assessment of left ventricular hypertrophy. 2D ECHO has recently been challenged by MRI and 3D echocardiography (13). ECG can substitute ECHO in LVH assessment in a setting where echocardiography is inaccessible. There are multiple ECG criteria for LVH with varying diagnostic yields. The American Heart Association (AHA) recommends testing the available ECG criteria in different populations of patients to identify ECG criteria with higher diagnostic yield (14).

A study done at the University of Sulaimani, Iraq, showed that the echocardiographic and ECG prevalence of LVH in hypertension patients is 30% and 16.5%, respectively (15). A different study in southwestern Nigeria found prevalence of the LVH by ECHO at 32.2% in 90 hypertensive participants. However, the prevalence of LVH by different voltage criteria in this study ranges from 13% to 45% (16, 17). There are very few published studies on the prevalence of LVH in hypertensive patients in Ethiopia. A study done in southwest Ethiopia found a prevalence of LVH by echocardiography in 200 hypertensives patients on treatment to be 52% (18–20).

Left ventricular hypertrophy is of clinical importance because LVH is evidence of target organ damage, an independent risk factor of cardiovascular and cerebrovascular events, and a very important factor in the risk stratification of hypertensive patients (8, 21). LVH is an important factor in the pathogenesis of ischemic heart disease, cardiac arrhythmias, congestive cardiac failure, and sudden cardiac death (21–23). Various studies have demonstrated regression of left ventricular hypertrophy with different interventions, i.e., antihypertensive therapy and the use of a regimen containing angiotensin-converting enzyme inhibitors (ACEI) (24, 25). Left ventricular hypertrophy in hypertensive patients needs to be detected and assessed promptly to prognosticate these patients, address modifiable risk factors such as obesity, and blood pressure, and the initiation therapy that has been shown to reverse LVH such as ACE inhibitors (26). Meta-analysis of clinical trials has shown that regression of LVH results in decreased risk of cardiovascular events (27).

LVH in hypertensive patients is important in risk stratification. Treatment of hypertension is based on individual risk categories in most guidelines. The European Society of Hypertension and European Society of Cardiology (ESH/ESC) 2018 guidelines risk stratifies hypertensive patients with LVH as “high-risk.”

Despite a high public burden in most low- and middle-income countries, there are few studies in Africa, particularly Ethiopia. There was no previous study done in the study area regarding the prevalence and associated factors among hypertensive patients with LVH. Therefore, this study aimed to show the prevalence and factors associated with LVH among patients with hypertension attending treatment in eastern Ethiopia.

## Methods and materials

### Study setting, design, and period

Hospital-based cross-sectional study design was employed at two public hospitals of Harar city in the Harari region from 1 January 2024 to 30 January 2024. Harar, one of Ethiopia's oldest cities and a UNESCO World Heritage site, is located 526 km east of Addis Ababa. The study sites, Hiwot Fana Specialized University Hospital (HFSUH) and Jugal Hospital, provide inpatient and outpatient services, including chronic disease management. These hospitals serve as teaching institutions for Haramaya University and cater to patients from Harari region, Eastern Hararghe, and the Somali Region (28).

### Source population, study population, and eligibility criteria

All adult hypertensive patients who have followed up at medical referral clinics of two public hospitals from 20 December 2021 to 20 December 2023. All adult hypertensive patients over 18 years of age were on follow-up at two public hospitals and with echocardiographic evaluation during the study period. The study excluded pregnant women with hypertension who were on follow-up at the obstetric ward and patients with an established diagnosis of structural heart disease before a diagnosis of LVH. Additionally, other potential causes of LVH, such as hypertrophic cardiomyopathy and aortic stenosis, were explicitly excluded to ensure that the observed LVH cases were primarily associated with hypertension.

### Sample size determination and sampling procedure

The sample size was calculated using a single population proportion formula with the following assumption: 95% confidence level, 5% margin of error, and 39.5% proportion of assuming prevalence of LVH in a recent study conducted at Ayder Comprehensive Specialized Hospital, Ethiopia, and adding 10% contingency for non-response rate, finally, 300 study participants were obtained.

The two public hospitals were selected for this study because they are the largest government hospitals in the area, with the highest patient volume. The patient registry logbook at the follow-up clinic was consulted to identify potential study participants. All medical record numbers of patients diagnosed with hypertension were collected, and their corresponding medical charts were retrieved. The inclusion criteria focused on hypertensive adult patients aged 18 years and older, who had a documented echocardiography.

During the study period, a total of 2,514 hypertensive patients were recorded—1,344 at Hiwot Fana and 1,170 at Jugal Hospital. The calculated sample size of 300 was proportionally allocated between the two hospitals, with 160 charts selected from Hiwot Fana and 140 from Jugal Hospital. However, because the study focused on echocardiographic evaluation, a total of 498 patient records containing echocardiographic data were identified—266 from Hiwot Fana and 232 from Jugal Hospital. After screening, 212 charts were excluded for not meeting the inclusion criteria. In the end, the final sample consisted of 264 patient charts, with 141 from Hiwot Fana and 123 from Jugal Hospital.

Charts were selected consecutively using a convenience sampling technique until the required sample size was reached.

### Data collection tools and procedure

#### Data collection instrument

Data were collected using a structured, pretested questionnaire and checklist to collect from patient charts (clinical data and radiologic report), and a structured questionnaire was used to extract data directly from the patient. The supervisor checked selected charts and assessed the abstracted data.

#### Data collection procedure

The chart review and patient interviews were conducted by five data collectors, consisting of three medical interns (final-year medical students who had passed their 5th-year qualification examination and were attached to their internship program in the medical ward) and two nurses assigned to follow-up clinics at two public hospitals during the study period. The principal investigator also played a supervisory role throughout the process.

Before data collection began, a 1-day training session was provided by the principal investigator, which covered the study's objectives, methods of data collection (including patient chart extraction), and the inclusion and exclusion criteria for participants.

All study participants were fully informed about the study's purpose, and the informed consent process, which included clear explanations of confidentiality and consent, was guaranteed. The principal investigator and supervisors closely monitored all activities during the data collection period to ensure proper adherence to study protocols.

##### For imaging data collection

Echocardiography was the procedure of choice for identifying LVH and the gold standard for assessment of LVH. Echocardiography could also permit quantitation of LV mass, including the severity of LVH, and give important information about the etiology of LVH (such as aortic or mitral valve disease or hypertrophic cardiomyopathy). Patients with echocardiographic evaluation were included in the study's proper checklist and questionnaire.

### Operational definitions

Hypertension: presence of persistently elevated systolic blood pressure (SBP) ≥ 140 mmHg and/or diastolic blood pressure (DBP) ≥ 90 mmHg or use of an antihypertensive drug(s) and lifestyle modification in patients 18 years of age and above (1, 29, 30).

LVH: defined when transthoracic echocardiographic left ventricular posterior wall thickness together with interventricular septal thicknesses is ≥11 mm (31, 32).

• Mild LVH: 11–13 mm
• Moderate LVH: 14–16 mm
• Severe LVH: ≥ 16 mm

Adherence to physical activity: walking 30 min per day for 3–5 days per week, regularly going to the gym, or engaging in heavy work (33).

Comorbidity: the simultaneous presence of two or more diseases or medical conditions in a patient (34).

Current smoker: in this study, a participant who had smoked cigarettes in the past 28 days (35, 36).

Former smoker: a participant who had a history of smoking cigarettes in their lifetime but who had not reported smoking cigarettes for the past 28 days (35, 36).

Alcohol consumer—male patients who drank more than two units of alcohol/day and female patients who drank more than one unit of alcohol/day (4).

Fruit and vegetable eating habits: those participants who had a habit of eating vegetables and fruits for <1 day/week were categorized as none, 1–3 days/week, and 4–7 days/week (4).

Body mass index (BMI): (BMI <18.5) underweight, normal (BMI 18.5–24.9), overweight (BMI >25) (4).

Waist circumference (WC): patients were classified into three health risk categories following cutoffs recommended by WHO, low risk (men, WC = 93.9 cm or less; women, WC = 79.9 cm or less), increased risk (men, WC = 94.0–101.9 cm; women, WC = 80.0–87.9 cm), and high risk (men, WC = 102.0 cm or more; women, WC = 88.0 cm or more) (37).

Adherence: medication adherence status was assessed as “Do you not take medication due to forgetfulness?”, “Do you not take medication due to carelessness?”, “Do you stop medication when feeling worse?”, and “Do you stop the medication when feeling good?” Respondents who reported “no” were considered to have high adherence and respondents who had a report of “yes” and had 2–3 scores were classified as medium adherence and a score of one was designated as low adherence (38, 39).

Adherence to physical activity: the practice of walking for 30 min per day for 3–5 days per week and the practice of regularly going to the gym or engaging in heavy work (4).

Wealth index: participants self-report poor “if their monthly income was <2,000 birr,” medium “if their monthly income was between 2,000 and 5000 birr,” and rich “if their monthly income was above 5000 birr” (40).

### Data quality control

To ensure the quality of data, a well-designed data abstraction checklist was used. The data abstraction checklist was a pre-test conducted on 5% of the total calculated sample size 2 weeks before the actual data collection. The questionnaire was examined for its clarity, understandability, and simplicity. The clarity of language was checked. The collected data were reviewed and checked daily for completeness and clearness by the primary investigator.

### Data processing and analysis

Data were checked, coded, and entered into EpiData version 3.2 and analyzed using SPSS version 29 software. Data about patients' sociodemographic, behavioral, and clinical characteristics were collected, and LVH prevalence status was based on echocardiographic findings done during follow-up descriptive statistics including frequencies, percentages, ranges, and standard deviations were done and presented using tables and figures. Bivariable and multivariable logistic regression analyses were used to determine factors independently associated with uncontrolled hypertension. All significant variables on bivariable analyses were allowed to enter the multivariate logistic regression model. Adjusted odds ratio (AOR) (with a 95% confidence interval) was used to measure the strength of association and statistical significance was determined at *P* < 0.05.

## Results

**Figure 1:**
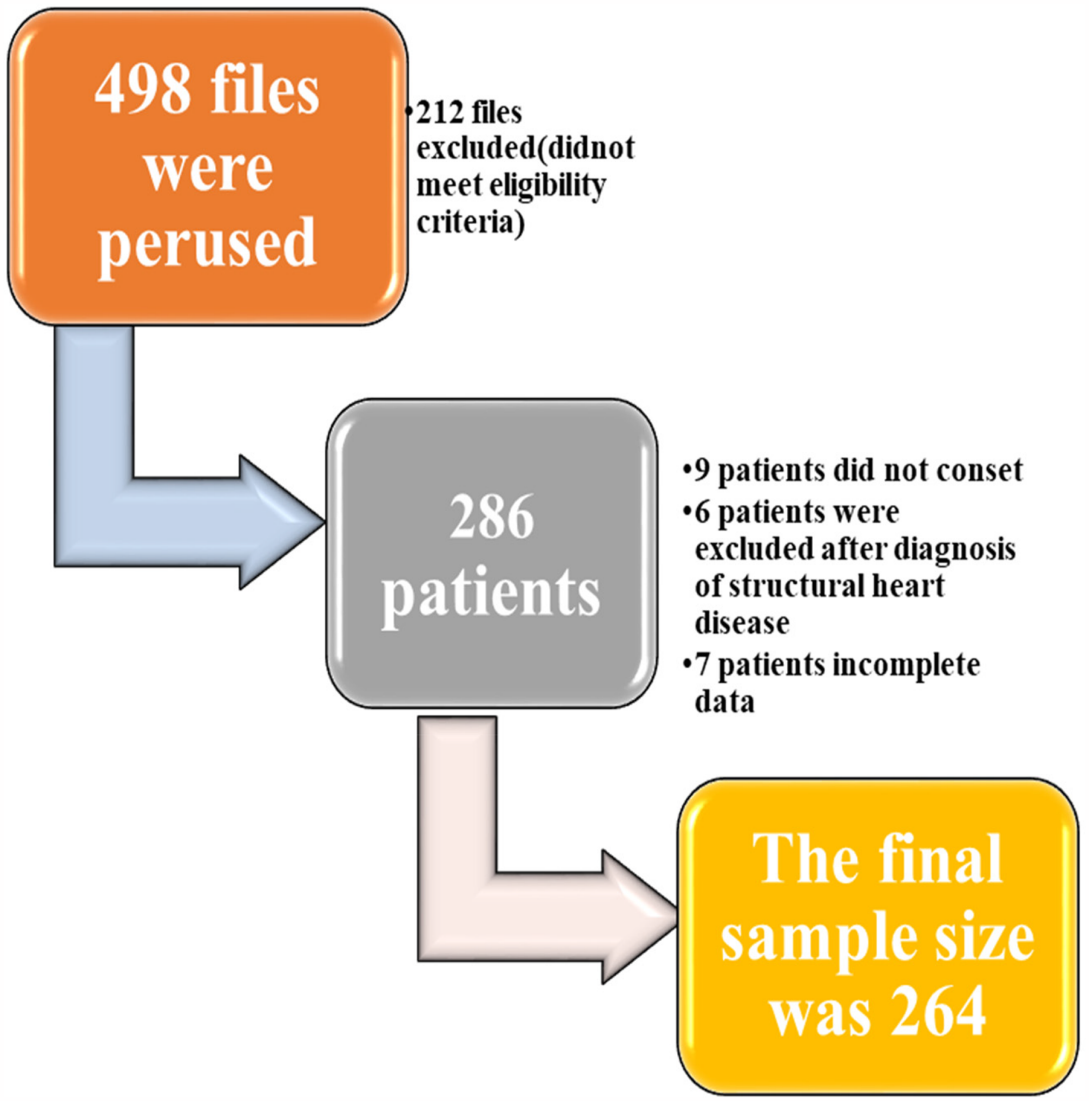
Flowchart for sample size determination for the study on prevalence and factors linked to left ventricular hypertrophy in hypertensive patients receiving care in Harari region, Ethiopia (2024).

The study was carried out among ambulatory patients at the medical outpatient clinic at HFSUH and Jugol Hospital from 20 December 2021 to 20 December 2023. A total of 498 patient files were screened, and 212 files were excluded. Two hundred sixty-four patients with a 92.3% retrieval rate were considered as the final sample size (Figure 1).

### Sociodemographic and clinical characteristics of respondents

#### Sociodemographic characteristics

Two hundred sixty-four hypertensive adult patients (>18 years old) on chronic follow-up care were included in the study. The majority of participants were in the age group of 50 and 65 years with a mean age of 58.4. More than half, 144 (54.5%), of participants were male. Concerning participants' education and the religion of respondents, 117 (443%) had no formal education, 158 (59.8%) were Muslim followers, and >80% were married (Table 1).

**Table 1:** Sociodemographic characteristics of adult hypertensive patients on follow-up at Hiwot Fana Specialized Compressive University Hospital and Jugol Hospital, Harar, Eastern Ethiopia, 2024 (*n* = 264).

Variables		Frequency	Percent
Sex	Male	144	54.5
	Female	120	45.5
Age	<60 years	144	54.5
	>60 years	120	45.5
Educational status	Unable to read and write	41	15.5
	Able to read and write	76	28.8
	Primary school	92	34.8
	Secondary school	41	15.5
	Collage and above	14	5.3
Occupation	Civil servant	25	9.5
	Merchant	54	20.5
	Farmer	64	24.2
	Housewife	72	27.3
	Self-employed	45	17.0
	Daily laborer	4	1.5
Marital status	Single	11	4.2
	Married	220	83.3
	Divorced	11	4.2
	Widowed	22	8.3
Place of residence	Urban	125	47.3
	Rural	139	52.7
Religion	Orthodox	79	29.9
	Muslim	158	59.8
	Protestant	24	9.1
	Catholic	3	1.1
Monthly income	<2,000	12	4.5
	2,000—5,000	89	33.7
	>5,000	163	61.7

#### Clinical characteristics

**Figure 2:**
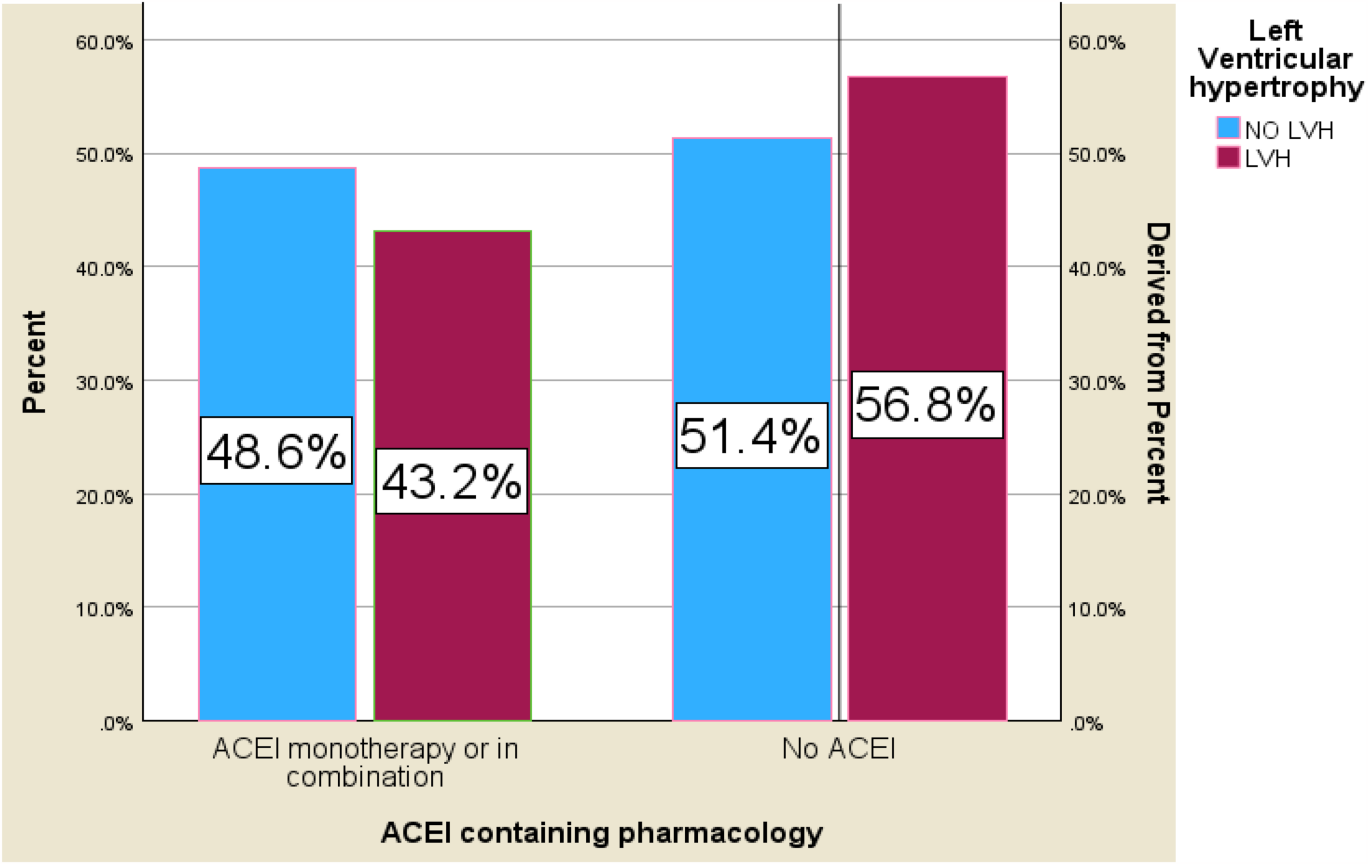
Bar chart for the prevalence of LVH and ACE inhibitor-containing medication among hypertensive patients on follow-up at HFSUH and Jugol Hospital, Eastern Ethiopia, 2024 (*n* = 264).

A total of 171 (65%) subjects with hypertension had been diagnosed for >10 years. Only 79 (30%) of participants had their blood pressure recorded at home. Seventy-eight (29.7%) of patients had been diagnosed with diabetes and were on glucose-lowering agents (insulin or oral glucose-lowering agent), and 64 (82%) of them had type 2 diabetes. Eighty percent of the diabetics had a mean fasting glucose level of >130 mg/dl. All of the participants were taking some type of antihypertensive agent at the time of the study. Approximately 46.3% of them were taking a combination of two or more antihypertensive drugs. The most commonly used drug was calcium channel blockers (CCBs) used in 64% of the participants as the only agent or in combination with others. Approximately 46.5% of them were taking angiotensin-converting enzyme (ACE) inhibitors alone or in combination with others. Thiazide diuretic alone or in combination with other agents was used by 33.3% of the participants. One hundred thirteen (43%) patients have a mean systolic measurement of >140 mmHg in two consecutive records. One hundred and twenty-two (46%) of the participants were taking two or more antihypertensive at the time of the study. Concerning participants' comorbidities, 15 (5.7%) patients had CKD, and 97 (30%) participants had dyslipidemia on treatment (Tables 2, 3, Figure 2).

**Table 2:** Clinical characteristics of adult hypertensive patients on follow-up at Hiwot Fana Specialized Compressive University Hospital and Jugol Hospital, Harar, Eastern Ethiopia, 2023 (*n* = 264).

Clinical variables		Frequency	Percent
Diabetes	Yes	78	29.5
	No	185	70.5
Types of diabetes	T1DM	14	17.9
	T2DM	64	82.1
Duration of diabetes in years	<10 years	10	12.8
	>10 years	68	87.2
Status of FBS Level	Controlled	15	19.2
	Uncontrolled	63	80.8
Dyslipidemia	Yes	97	36.7
	No	167	63.3
Chronic kidney disease	Yes	15	5.7
	No	249	94.3
Duration of hypertension	<10 years	93	35.2
	>10 years	171	64.8
Home BP measurement	Yes	80	30.3
	No	184	69.7
Initiation of antihypertensive at the time of diagnosis	Yes	79	29.7
	No	185	70.3
Mean systolic BP	<140	145	54.9
	>140	119	45.1
Mean diastolic BP	<90	164	62.1
	>90	100	37.9
Severity of hypertension at the time of diagnosis	>140/90	75	28.4
	>160/100	184	69.7
	>180/110	5	1.9

**Table 3:** Prescribing pattern of antihypertensive drugs among hypertensive patients on follow-up at HFSUH and Jugol Hospital, Eastern Ethiopia, 2023 (*n* = 264).

Variables	Prescribing drug pattern	Frequency	Percent
The number of antihypertensive drugs	Monotherapy	142	53.8
	Dual therapy	109	41.3
	Triple therapy	13	4.9
The types of antihypertensive group of drugs	ACEI	50	18.9
	CCBs	61	23.1
	Thiazide	24	9.1
	ARBs	6	2.2
	ACEI + CCBs	50	18.9
	ACEI + TZDS	14	5.3
	ACEI + BB	1	.4
	CCB + loop diuretics	3	1.1
	CCB + TZD	44	16.6
	CCB + BB	2	.8
	CCB + ARBS	1	.4
	ACEI + CCB + TZD	6	2.3
	ACEI + CCB + loop DIURETICS	2	.8
Commonly used monotherapy	Amlodipine	53	20.1
	Enalapril	53	19.3
	HCT	23	8.7
	Nifedipine	8	3
Commonly used dual therapy	Amlodipine + HCT	34	12.9
	Enalapril + amlodipine	46	17.4
	Enalapril + HCT	15	5.7
Triple therapy	Enalapril + HCT + amlodipine	8	3

#### Behavioral characteristics

Eighty-eight (36.4%) of the participants and 53 (20.1%) participants gave a history of smoking and alcohol consumption, respectively. Additionally, 139 (52.7%) patients chew chat. One hundred sixty-seven (63.3%) participants reported adding salt to their food. The overall prevalence of physical inactivity among participants was 226 (85.6%); out of these participants, 32% had left ventricular hypertrophy, and 33.3% had uncontrolled blood pressure. Thirty-seven (14%) of the participants had a family history of hypertension (Table 4).

**Table 4:** Behavioral characteristics of adult hypertensive patients on follow-up at Hiwot Fana Specialized University Hospital and Jugol Hospital, Harar, Eastern Ethiopia, 2024 (*n* = 264).

Variables		Frequency	Percent
Smoking	Yes	88	36.4
	No	176	63.6
Khat chewing	Yes	139	52.7
	No	125	47.3
Alcohol intake	Yes	53	20.1
	No	211	79.9
Adherence to medication	Yes	189	71.6
	No	75	28.4
Regular exercise habits	Yes	38	14.4
	No	226	85.6
Family history of HTN	Yes	37	14
	No	227	86
Salt reduction adherence	Adherent	97	36.7
	Non-adherent	167	63.3

#### Left ventricular hypertrophy status

**Figure 3:**
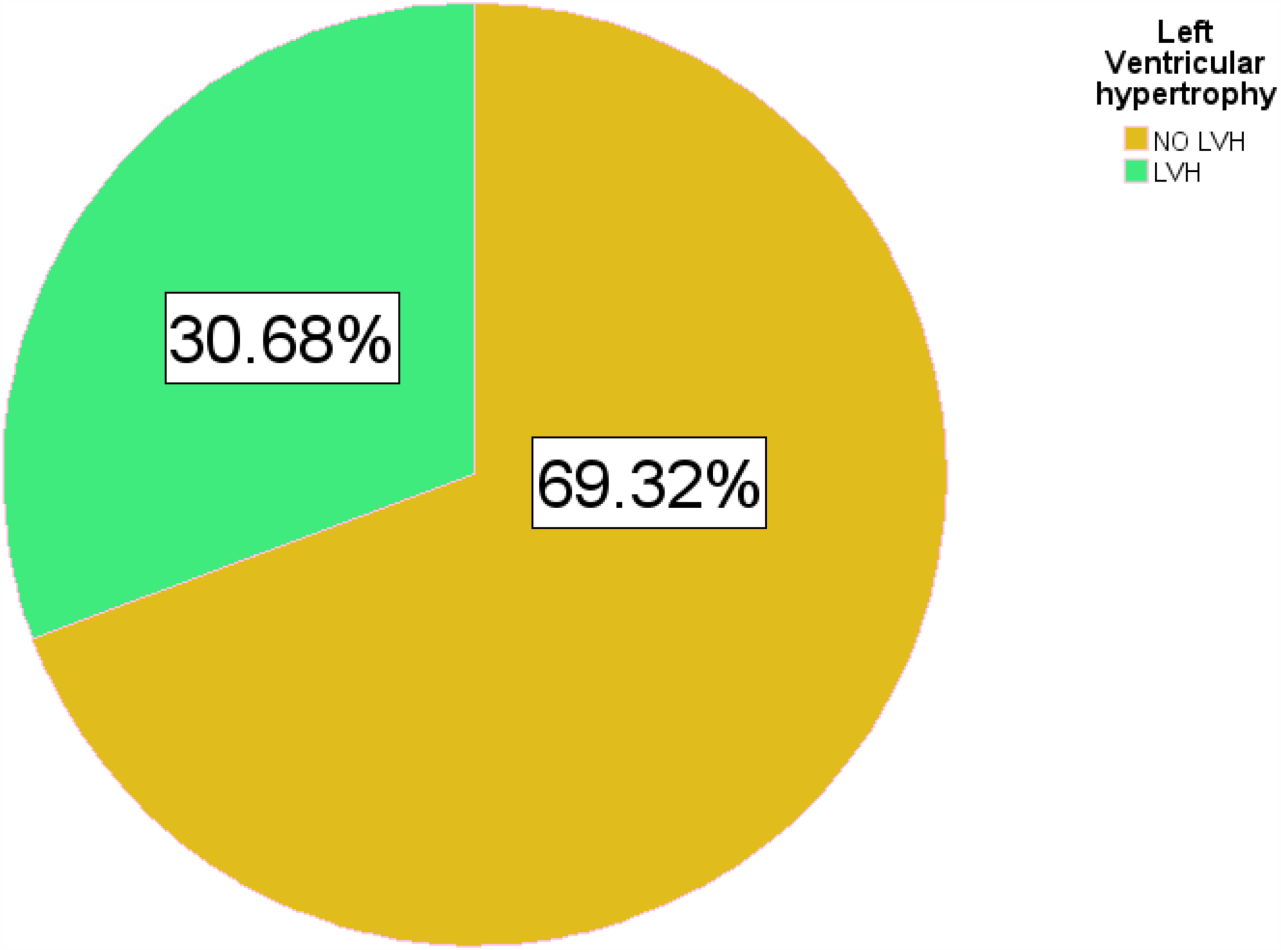
Pie chart for the prevalence of LVH among hypertensive patients on follow-up at HFSUH and Jugol Hospital, Eastern Ethiopia, 2024 (*n* = 264).

The overall prevalence of echocardiographic LVH was 30.7% (95% CI: 25.1%–36.3%). The majority of the patients had mild LVH at 42 (51.8%). The mean posterior wall thickness in diastole (PWTd) and interventricular septal wall thickness in diastole (IVSTd) were 10.6 and 10.13 mm, respectively (Table 5, Figure 3).

**Table 5:** Summary of echocardiography findings of the study of adult hypertensive patients on follow-up at Hiwot Fana Specialized University Hospital and Jugol Hospital, Harar, Eastern Ethiopia, 2024 (*n* = 264).

Variables		Frequency	Percent
Left ventricular hypertrophy	No LVH	183	69.3
	LVH	81	30.7
Severity of LVH	No LVH	183	69.3
	Mild LVH	42	15.9
	Moderate LVH	33	12.5
	Severe LVH	6	2.3
Interventricular septal wall thickness in diastole (IVSTd) in *mm*	Mean	10.13	
	Minimum	7	
	Maximum	18	
LV Posterior wall thickness in diastole (PWTd) in *mm*	Mean	10.60	
	Minimum	8	
	Maximum	21	

#### Risk factors for the development of left ventricular hypertrophy

To identify risk factors associated with left ventricular hypertrophy, both bivariate and multivariate ordinal logistic regression analysis was performed. To determine candidate variables, a bivariate logistic regression was performed on all symptomatic cases (*N* = 264) by including sociodemographic, behavioral, and clinical characteristics at presentation and echocardiographic findings at follow-up as independent variables. Continuous variables were transformed into categorical variables. Analysis was performed for each variable at a significance level of 0.25. The result from bivariate regression showed that sex, age group older than 60 years, diabetes mellitus, duration of hypertension, home BP recording, adherence to the medication, cigarette smoking, khat chewing, salt reduction adherence, the number of antihypertensive medications used, CKD, dyslipidemia, and mean systolic and diastolic BP recording were identified to be significantly associated with the development of LVH (*p* < 0.025).

To determine independent risk factors associated with LVH, a multivariate ordinal logistic regression was done at a significance level of 0.05 by including all variables associated with significant *p*-value (<0.05) in bivariate analysis. After adjusting for other factors, the result of multivariate analysis showed that age older than 60 years, diabetes mellitus, adherence to antihypertensive medications, uncontrolled hypertension, and duration of hypertension were significantly associated with the risk of developing LVH (*p* < 0.05).

Among the 81 patients with left ventricular hypertrophy (LVH), 68 (83.9%) were aged 60 or older, making individuals over 60 six times more likely to develop LVH than those under 60 (AOR = 5.981, CI = 1.832–19.522). A history of khat chewing increased the risk of LVH nearly threefold (AOR = 2.676, CI = 1.786–9.109), while diabetes mellitus was associated with a 10-fold higher risk compared to non-diabetic individuals (AOR = 10.430, CI = 2.904–37.454). Non-adherence to antihypertensive medications also significantly raised the odds of LVH (AOR = 4.132, CI = 1.208–14.141). Similarly, uncontrolled systolic blood pressure increased the risk eightfold (AOR = 8.340, CI = 2.280–30.512), and the absence of home blood pressure monitoring was associated with nearly five times the risk of LVH (AOR = 5.591, CI = 1.041–30.012).

Hypertension duration also emerged as a critical factor. Approximately 55% of participants had hypertension for 10 years or more, with a markedly higher prevalence of LVH (51.7%) compared to those diagnosed <5 years ago (5.1%). After adjusting for potential risk factors, individuals with hypertension lasting 10 years or more were eight times more likely to develop LVH than those with a shorter duration (AOR = 8.766, CI = 2.101–36.584) (Table 6).

**Table 6:** Multivariable logistic regression of factors associated with the development of LVH among hypertensive patients in Harar Town, Eastern Ethiopia, 2024 (*n* = 264).

Associated factors		Left ventricular hypertrophy		AOR (95%)	*p*-value
		No LVH	LVH		
Sex	Male	131 (64.5)	51 (35.5)	1.258 (0.373–4.239)	0.711
	Female	90 (75)	30 (25)	1	
Age	<60 years	131 (90.9)	13 (10.1)	1	
	>60 years	52 (43.3)	68 (56.7)	5.981 (1.832–19.522)^b^	0.003
Khat chewing	Yes	85 (61.1)	54 (38.9)	2.676 (1.786–9.109)^b^	0.001
	No	98 (78.4)	27 (21.6)	1	
Smoking	Yes	50 (56.8)	38 (43.2)	1.169 (0.324–4.216)	0.811
	No	133 (75.5)	43 (24.5)	1	
Salt reduction adherence	Adherent	87 (89.6)	10 (10.4)	1.738 (0.292–10.349)	0.544
	Non-adherent	96 (57.4)	71 (42.6)	1	
Diabetes	Yes	21 (26.9)	57 (73.1)	10.430 (2.904–37.454)^b^	<0.001
	No	162 (85.7)	24 (14.3)	1	
Home BP measurement	Yes	72 (90)	8 (10)	1	
	No	111 (60.3)	73 (39.7)	5.591 (1.041–30.012)^a^	0.045
Dyslipidemia	Yes	54 (55.6)	43 (44.4)	.830 (0.240–2.870)	0.768
	No	129 (77.2)	38 (22.8)	1	
The number of antihypertensive drugs	Monotherapy	124	17	1	
	Two or more drugs	59	64	1.014 (0.314–3.277)	0.982
Adherence of medications	Adherent	124 (83.2)	25 (16.8)	4.132 (1.208–14.141)^a^	0.024
	Not-adherent	19 (25.3)	56 (74.7)	1	
Mean systolic BP	<140	135 (93.1)	10 (6.9)	1	
	>140	48 (40.3)	71 (59.7)	8.340 (2.280–30.512)^b^	0.001
Mean diastolic BP	<90	141 (85.9)	23 (14.1)	1	
	>90	42 (42)	58 (58)	3.242 (.740–10.104)	0.053
Duration of hypertension	<10 years	113 (94.9)	6 (5.1)	1	
	>10 years	70 (48.2)	75 (51.8)	8.766 (2.101–36.584)^b^	0.003

CI, confidence interval; COR, crude odds ratio; AOR, adjusted odds ratio.

^a^ *p* < 0.05.

^b^ *p* < 0.005.

## Discussion

The primary objective of this study was to investigate the prevalence of LVH status and associated factors. A total of 264 hypertensive patients were included in this hospital-based cross-sectional study. LVH is an independent cardiovascular risk factor (41). Its prevalence in hypertensive patients in most literature worldwide varies from 20% to 70% based on the population studied and the criteria used. In the current study, the prevalence of LVH among hypertensive patients on outpatient follow-up was 30.7% (95% CI: 25.1%–36.3%). The result of this study was consistent with cross-sectional studies done on the echocardiographic prevalence of left ventricular hypertrophy among hypertensive patients in a tertiary health institution in Nigeria and Iran which was 32.4% (42) and 30% (15), respectively. The result of this study was higher than that of a study done for prevalence and risk factors of abnormal left ventricular geometrical patterns in untreated hypertensive patients in China which shows a prevalence LVH of 20.2% (43). The prevalence of LVH reported in this study is lower than that in a study conducted in Jimma, Ethiopia (18); Aksum, Ethiopia (44); and Basel, Switzerland (45). This inconsistency could be due to the operational definition of LVH used and diagnostic method in our study and other respective studies.

**Figure 4:**
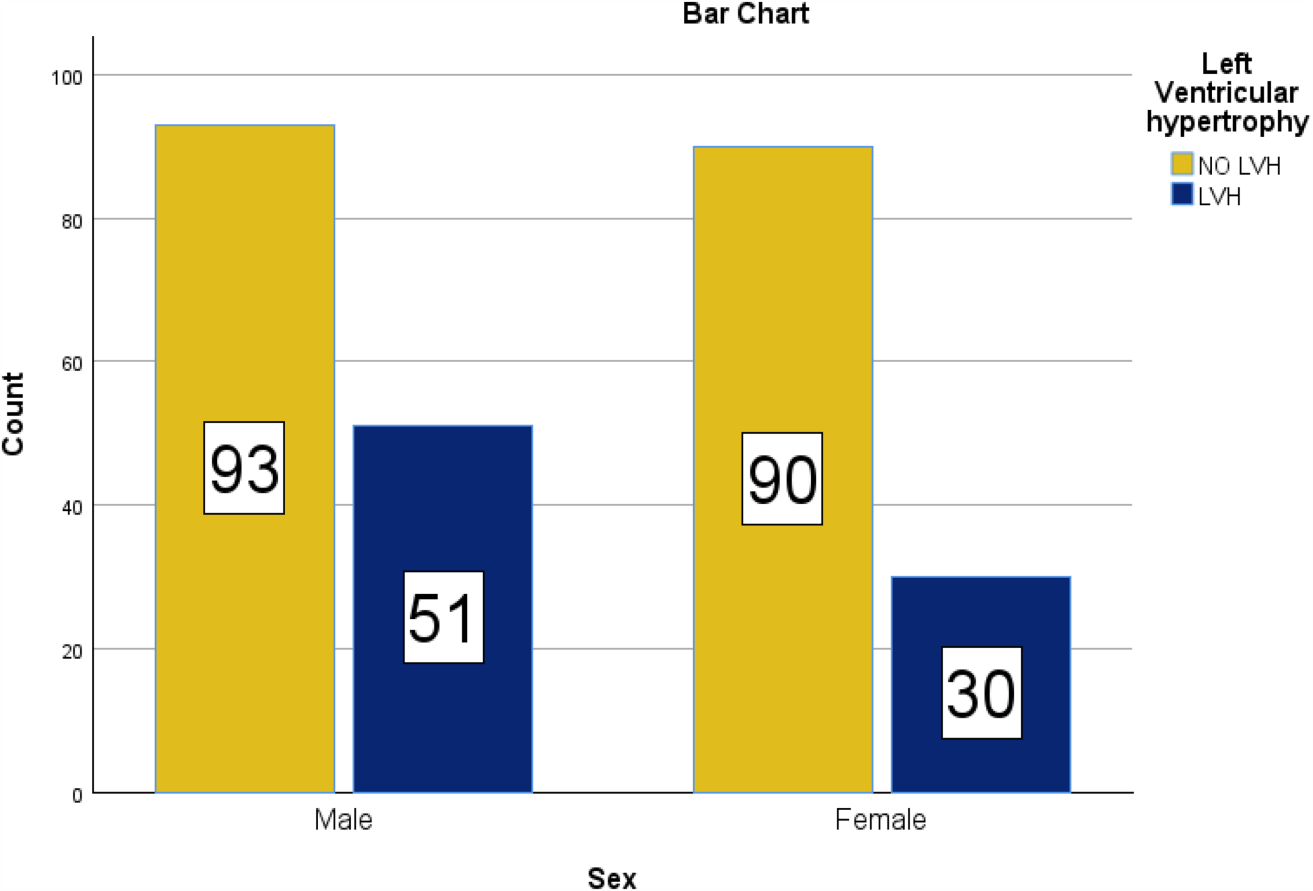
Bar chart for the sex distribution in the prevalence of LVH among hypertensive patients on follow-up at HFSUH and Jugol Hospital, Eastern Ethiopia, 2024 (*n* = 264).

Age was found to be an independent risk factor for LVH in most studies. Age by itself is a great predictor of cardiovascular disease. Increasing age makes cellular, structural, and functional changes in the heart. These maladaptive changes are more accentuated in older adults with a high degree of frailty (46). We found very similar findings in our series, with LVH becoming more pronounced after 60 years of age (Figure 4). History of khat chewing was found to have a significant independent risk factor for LVH in our study, that is, khat chewing results in many medical complications, particularly in the cardiovascular system, and this may be due to the chronic stimulant effects of khat use which may cause sustained hypertension and contribute to LVH over time.

Non-adherence to antihypertensive medication was also positively associated with the development of LVH. More than half, 63%, of the study subjects in HFSUH and Jugol Hospital with low to medium adherence to antihypertensive medication had uncontrolled blood pressure. This might be explained as 38.2% of the participants had poor/medium low income, and those with health insurance coverage for medications missed at least one dose in the 12 months due to stockouts and inability to afford medications outside of insurance coverage.

The presence of diabetes has shown significant correlation with LVH in most studies (47). Similarly, the NOMAS cohort study conducted among a multiethnic population in the USA, showed that DM was independently associated with an increased risk of LVH (48). The finding in our study is also consistent with this finding, as diabetes was found to be an independent risk factor for LVH in hypertensive patients with an AOR of 10.

Our finding reported that the prevalence of LVH among males was not significantly higher than that identified among females. There are too many controversies regarding the relationship between gender and LVH. There are studies that showed that females have a positive association with LVH (15, 43). However, other studies confirmed the reverse of this (49). The prevalence of LVH found in men and women with essential hypertension is strikingly dependent on whether one uses such sex-specific criteria or a single criterion of LV mass indexed for body surface area (50). Whether these differences are due to the population studied or the criteria or cutoff points used needs to be addressed in future studies.

Duration of hypertension was found to be an independent predictor of left ventricular hypertrophy in our study which is consistent with most studies from around the world (51). More than 90% of patients with LVH in this study had a duration of hypertension of >10 years.

Uncontrolled systolic blood pressure ≥140 mmHg and diastolic BP >140 mmHg were associated with the development of left ventricular hypertrophy in this study with adjusted OR of 3.7 and 3, respectively. Eighty percent of patients with LVH had a mean systolic BP of >140 mmHg, and 70% of patients had a mean diastolic BP of >90 mmHg; in addition, only 30% of the participants had BP recording at home. This is also consistent with most other studies (42).

There are also too many controversies regarding the relationship between antihypertensive medication and the development of LVH. ACE inhibitors were associated with the reduction in the occurrence of LVH and promoting regression of LVH after long-term treatment (18, 52). The other meta-analysis from China shows amlodipine treatment in patients with hypertension significantly reduced the LV mass index and LV posterior wall thickness, without notably affecting the LV end-diastolic diameter (53). The other meta-analysis study revealed a matched comparison of renin–angiotensin system inhibitors (RASi) showing that the effect of ACEI in reducing left ventricular mass index (LVMi) was not as effective as that of angiotensin receptor blockers (ARBs). ARBs were more effective among the different types of antihypertensive drugs (27). Lower LVH prevalence was seen in patients taking ACE inhibitors alone or in combination with other agents at the time of our study. However, this was not statistically significant.

### Limitations

The participants were those on long-term pharmacological treatment for hypertension, and this might underestimate the prevalence of LVH. There might be bias since the behavioral characteristics of the participants were based on self-report. There was also a limitation on the wealth index assessment parameter used in this study. Since the study was an echocardiography-based prevalence of LVH, the major limitation of this study was that transthoracic echocardiography should not be done by radiologists and gender-based LVM index measurement should be applied to determine the presence of LVH. The study enrolled only 264 participants, falling short of the calculated sample size of 300. This discrepancy limited our ability to use a probability sampling technique and likely contributed to increased variability, resulting in reduced precision and a wider confidence interval. The short period used for data collection also contributed to the problem.

## Conclusions

The prevalence of echocardiography-documented LVH in the two public hospitals (HFSUH and Jugol Hospital) was 30.7%. Age, duration of hypertension, poor adherence to medication, uncontrolled hypertension, and history of diabetes were found to be independent predictors of LVH in this study. To effectively understand the prevalence and associated factors of LVH in hypertensive patients in our country, a further collaborative study at a regional and national level with a large sample and diverse group of patients is required. The cardiologist should be the one supposed to do the echocardiographic exam. The HFSUH management is expected to have at least one cardiologist in the town. Counseling hypertensive patients on drug adherence, home BP measurement, doing moderate physical activity, and avoiding salt addition habits in meals are recommended to improve BP control and prevent LVH in hypertensive patients at follow-up clinics.

## Data Availability

The original contributions presented in the study are included in the article/Supplementary Material, further inquiries can be directed to the corresponding author.
